# The Midwives Bill

**Published:** 1902-06-28

**Authors:** 


### EDITOR'S LETTER-BOX.

### THE MIDWIVES BILL.

J. WILSON, President, Incorporated Midwives Institute, writes: I venture to think that your leader in THE HOSPITAL of the 14th inst. on the Midwives Bill is scarcely fair to the attitude assumed by present and former Governments to what you justly term "this greatly needed measure."

While the public remained apathetic towards the needless loss of life amongst poor women, the medical profession with some honourable exceptions, devoted much of their strength to opposing legislation; and as a rule, where the medical press was not critical, it was hostile. Under these circumstances, it is difficult to conceive how any Government could assume a strong attitude.

As late as last November, at the election of direct representatives to the General Medical Council, the two candidates who were elected expressly stated that they were opposed to legislation for midwives in any form. The result of that election proves how large a section of general practitioners are still opposed to the principle of the Bill.

In your leader you say that "in the many years which have passed since the state of midwifery practice in England was shown to be a public scandal, urgently calling for legislative treatment, hundreds of lives must have been lost as a direct result of the indifference of our representatives to any question in which nothing more serious than the avoidable mortality of poor women in childbirth was involved. Time after time, session after session, some progress has been made with a greatly needed measure, but no Government has yet felt the care of such a measure to be a duty."

In the past twelve years many Bills have been drafted and referred to the General Medical Council for advice and assistance; but while the Government was assured that the question was one that closely concerned the medical profession, that profession gave no united support in favour of legislation, offered little more than criticism, and not unfrequently provided active and positive opposition. But for this fact, a Midwives Bill would doubtless have become law some time ago, many of the preliminary difficulties attendant on setting the Act in motion would by this time have been overcome, and in consequence much needless suffering and loss of life have been prevented.

It is, I think, generally acknowledged that no Government however strong is particularly eager to give advantages to "contentious" measures, especially those which affect vested interests; only a strong and determined demand from influential and united bodies can, as a rule, command attention on such questions. Legislation regarding midwives can, I fear, scarcely be considered to fulfil the latter conditions.

The gradual creation of a healthy public opinion, largely due to the influence of the Society for Compulsory Registration of Midwives, supported by the invaluable assistance of a few eminent medical men, has at last drawn attention to the vital importance of this measure.

It should be remembered that the section of the poorer community affected by the Bill is one which is practically without representation, and which should therefore at least receive adequate protection.

[We cannot admit that our remarks were in any way unfair. Miss Wilson's letter is interesting as showing the motives by which she considers that Governments are influenced, but there is nothing in it in any way to controvert, and indeed there is much to support, what we said—namely, that no Government had felt the care of such a measure as the Midwives Bill to be a duty. Not only is this true, but even now it remains the fact that the chief risk to the efficiency of the present Bill lies in the danger lest the influence of the Home Secretary should still be brought to bear to wreck the particular clause in it which he dislikes, a clause without which the Bill must be absolutely useless for its only legitimate purpose—that, namely, of preventing needless loss of life amongst poor women. It undoubtedly lies with the Government at the present moment to say whether the Bill shall be allowed to pass in an effective form as it left the House of Commons, or shall be reduced again to the position of a mere popularity-hunting and useless measure. If it be true, as has been so often said and as we believe, that the lives of many women every year depend upon a stop being put to the practice of midwifery by ignorant persons, the lives of these women must undoubtedly lie at the door of the Government if at this stage it should intervene to delete from the Bill the only clause by which the continuance of the practice alluded to is rendered illegal. We shall see what the Government will do.—ED. THE HOSPITAL.]

